# Systematic review on the association of COVID-19-related conspiracy belief with infection-preventive behavior and vaccination willingness

**DOI:** 10.1186/s40359-022-00771-2

**Published:** 2022-03-15

**Authors:** Tilli Ripp, Jan Philipp Röer

**Affiliations:** Department of Psychology and Psychotherapy, Witten/Herdecke University, Alfred-Herrhausen-Straße 50, 58455 Witten, Germany

**Keywords:** Conspiracy belief, Conspiracy theory, Infection-preventive behavior, Containment-related behavior, COVID-19, Coronavirus, Vaccination, Vaccination willingness, COVID-19 pandemic

## Abstract

**Background:**

In times of a pandemic, not only infections but also conspiracy narratives spread among people. These have the potential to influence the course of the pandemic. Here we summarize and critically evaluate studies from the first year of the pandemic presenting findings on the association between COVID-19-related conspiracy belief and infection-preventive behavior and vaccination willingness.

**Method:**

A systematic literature search was conducted using the databases *COVID-19 Data Portal, APA PsycArticles, Psychology and Behavioral Sciences, Scopus*, and *PubMed*. After removing duplicates, studies meeting the previously defined inclusion and exclusion criteria were subjected to title and abstract screening and content reviewed and analyzed subsequently.

**Results and conclusion:**

The systematic literature search yielded 17 studies meeting our pre-specified inclusion criteria. Twelve studies examined infection-preventive behavior (*N* = 16,485), and ten studies vaccination willingness (*N* = 20,210). In summary, belief in COVID-19-related conspiracy narratives was negatively associated with vaccination willingness and infection-preventive behavior. The results point to the importance of the content of the conspiracy narratives. Various explanatory approaches and possible moderator variables are discussed, referencing the state of research on conspiracy beliefs and health-related preventive behavior after the first year of the pandemic. We argue that future studies should strive for a consistent operationalization and use of the term conspiracy belief.

**Supplementary Information:**

The online version contains supplementary material available at 10.1186/s40359-022-00771-2.

## Background

Crises of historic proportions such as the COVID-19 pandemic are typically accompanied by heightened collective uncertainty and fear [1]. In this situation, belief in conspiracy narratives can provide orientation and security. A particularly large number of conspiracy narratives revolve around historical events such as pandemics, as well as terrorist attacks, which can be explained not only by the insecurity they create and the feeling of a lack of control among the population, but also by the fact that events that are perceived as collectively threatening on the one hand and as significant on the other are particularly suitable as a basis for conspiracy narratives [2, 3]. Between 1918 and 1919, when the Spanish flu was rampant, and in 2009, during the outbreak of the so-called swine flu (H1N1), an increase in conspiracy belief among the affected population was observed [4, 5]. Likewise during the COVID-19 pandemic, conspiracy narratives circulated within a short period of time. They vary in detail and abstractness and differ in terms of content, for example addressing the origin, characteristics or spread of the virus, targeting specific actors and their interests, or doubt the existence of the coronavirus. Conspiracy narratives have the potential to influence the course of the crisis that initially fostered their occurrence. This is because effective control of the highly contagious virus requires widespread public adoption of preventive behavior [6] and vaccination of a high proportion of the population [7]. The negative association between conspiracy belief and health-related preventive measures has already been demonstrated in various research projects [8–11]. People who believe in conspiracies where not only found to be less likely to attend annual checkups, visit the dentist, and use sunscreen [12], according to Jolley and Douglas [13], they are also less likely to support public action on pandemics. Furthermore, conspiracy belief correlates with concerns towards vaccination and lowers vaccination willingness [12, 14, 15]. Similar effects might be expected with respect to COVID-19. To control the ongoing COVID-19 pandemic, effective vaccines are an essential tool because they can be critical in reducing transmission, hospitalizations, and the need for intensive care [16, 17]. However, the hoped-for indirect community protection, known as herd immunity, can only occur if enough people are willing to be vaccinated [18]. Until herd immunity is achieved, infection prevention measures remain critical to contain the pandemic [19]. This paper therefore examines the relationship between belief in COVID-19-related conspiracy narratives and infection-preventive behavior and vaccination willingness. Since the studies dealing with this question were conducted at different points in time and the samples considered vary to a large extent and there is no uniform operationalization of the constructs, comparability is significantly hampered. Therefore, a systematic examination of similarities and differences is necessary to reliably determine the state of research.

## Method

### Definition of the surveyed factors

In international psychological and social science research, there is conceptual disagreement in analyzing conspiracies. Commonly, the terms conspiracy theory and conspiracy belief are used. According to Douglas et al. [20, p. 4] conspiracy theories can be defined as “attempts to explain the ultimate causes of significant social and political events with claims of secret plots by two or more powerful actors”. Instead of the misleading term conspiracy theory, which seems to suggest a scientific theoretical background, we decided to use the term conspiracy narrative in our review. Belief in conspiracy narratives is referred to as conspiracy belief. The term COVID-19-related conspiracy belief refers to belief in conspiracy narratives that relate to the COVID-19 pandemic. Any behavior that contributes to reducing the spread of COVID-19 and thus to pandemic containment is subsumed under the term infection-preventive behavior. On the one hand, this includes the implementation of hygiene measures such as hand washing or hand disinfection, adherence to cough and sneeze etiquette, avoiding touching one's face in public, and cleaning surfaces. On the other hand, this includes measures of social distancing or physical distancing such as staying at home, keeping a distance, limiting contact, avoiding crowds, and avoiding contact with sick or elderly people. Also included are specific measures such as wearing a mask or gloves, staying home with symptoms of illness, using testing services, avoiding travel and using a contact-tracking app. Vaccination willingness refers to the expressed intention to be vaccinated against COVID-19 if vaccination is available. The vaccination hesitancy variable is understood to be the opposite of vaccination willingness.

### Literature search procedure

**Fig. 1:**
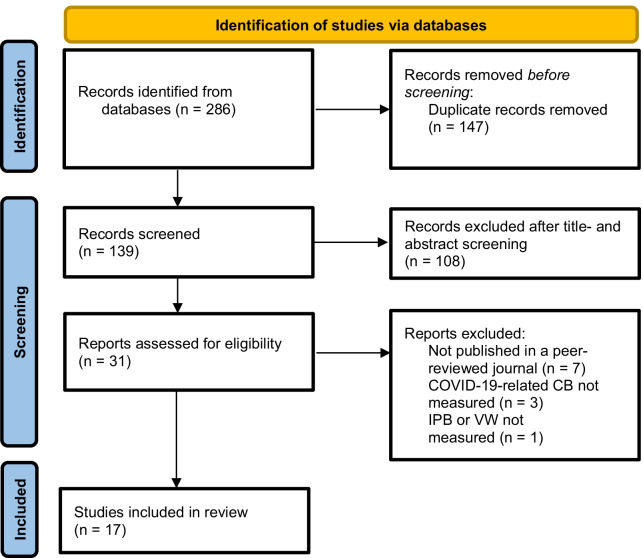
Flow diagram on study selection. *CB* conspiracy belief, *IPB* infection-preventive behavior, *VW* vaccination willingness

In the first step the search terms for the literature search were determined. To identify relevant articles related to the prevailing COVID-19 pandemic, the search terms COVID-19 and coronavirus were used. For coverage of studies dealing with beliefs in conspiracy narratives, the keywords conspiracy theories and conspiracy beliefs were used. To identify appropriate studies to answer the research question, the final search term was created by linking the search terms ((conspiracy theories OR conspiracy beliefs) AND (COVID-19 OR coronavirus)) and conducting a systematic literature search on February 3, 2021 using the databases *COVID-19 Data Portal, APA PsycArticles, Psychology and Behavioral Sciences, Scopus*, and *PubMed*. Duplicates were removed in the following step. The search resulted in a varying number of matches in the databases (see Additional file 1: Table A1). Using the previously defined inclusion and exclusion criteria, the remaining studies were subjected to title and abstract screening and finally reviewed for content. In total, 17 studies were extracted through the described search process and with the help of the inclusion and exclusion criteria (see Additional file 1: Table A2), which form the research basis of the present paper. Figure 1 shows the systematic search process, including the results obtained in each step.

### Inclusion and exclusion criteria

Only articles with data collected and published during the COVID-19 pandemic were included (i.e., studies that collected and published their data between March 11, 2020 and February 2, 2021) [21]. To ensure the quality of the selected studies, a further requirement was publication in a peer-reviewed journal. Preprints were excluded. To increase the comparability and generalizability of the data, only quantitative, empirical studies that investigated an adult sample were included. The included studies measured COVID-19-related conspiracy beliefs as well as infection-preventive behavior and/or vaccination willingness, according to the definition established in advance (see Definition of the surveyed factors). Only studies in English were included and only those studies that reported statistical analyses about the association between COVID-19-related conspiracy beliefs and infection-preventive behavior and/or between COVID-19-related conspiracy beliefs and vaccination willingness.

## Results

The total sample size of the twelve studies examining infection preventive-behavior (see Table 1) is *N* = 16,485, with the smallest sample counting *N* = 407 participants [22], the largest sample counting *N* = 2501 participants [23], and the average age ranging from 31 years [24] to 47 years [23]. The participants come from different nations. Two studies examined a Polish sample [25, 26], and one sample each was collected in Serbia [22] and Turkey [24]. The majority of the studies was conducted in English-speaking countries: Three studies each examined U.S. samples [27–29], and UK samples [23, 30, 31]. In addition, there is a study with samples both from the US and UK [32], and an international sample with participants from 66 nations, but with the majority also from the US or UK [33]. Conspiracy belief was measured in a variety of ways. For example, Alper et al. [24] used two items that asked about a conspiratorial plan behind the spread of coronavirus, while Freeman, Waite, et al. [23] used 48 items to cover the belief in a wide variety of conspiracy narratives. Imhoff and Lamberty [32] used two sets of conspiracy narratives with three items each: COVID-19 is a hoax and SARS-CoV-2 is human-made. Oleksy et al. [26] distinguished between general and government-related conspiracy narratives. To assess infection-preventive behavior, the studies reviewed varied in the number of examined behaviors that reduce the spread of COVID-19. In some cases, these were defined following government or WHO recommended guidelines or health recommendations [22, 23, 25, 29]. Mostly, this included hygiene measures and social distancing measures. Bierwiaczonek et al. [27] limited their survey to social distancing measures and Freeman, Waite, et al. [23] additionally examined the use of COVID-19-related medical services. Biddlestone et al. [33] surveyed hygiene measures and social distancing separately. Studies reported either correlation coefficients [22, 23, 34, 35], odds ratios [23, 28, 36–38], standardized regression coefficients [29, 34], or unstandardized regression coefficients [22, 28, 31]. Biddlestone et al. [33] created a standard equation-model (SEM) model, Bierwiaczonek et al. [27] worked with a crossed-lagged panel model, and Romer et al. [29] created a path model for the association of variables between the first and second measurement. Ten of twelve of the reviewed studies demonstrated a negative correlation of conspiracy beliefs and infection-preventive behavior. The smallest correlation reported was 0.16 [33], and the largest was − 0.524 [32]. The various regression analyses yielded odds ratios ranging from 0.37 [30] to 14.34 [23], standardized regression coefficients from 0.36 to 0.38 [31], and unstandardized regression coefficients from 0.04 [26, 28] to 0.834 [32]. In the SEM model of Biddlestone et al. [33], conspiracy belief emerged as a negative predictor of social distancing: *β* = − 0.04, *p* < 0.001. In the crossed-lagged panel model of Bierwiaczonek et al. [27], conspiracy belief at an earlier measurement time point predicted social distancing at the following measured time point: *β* (− 0.062 to − 0.067), *p* = 0.002. Alper et al. [24] and Earnshaw et al. [28] found no significant association between conspiracy belief and infection-preventive behavior. The difference in the strength of the association is striking, depending on the content of the conspiracy narratives on which the conspiracy belief variable is based. For example, general COVID-19-related conspiracy beliefs were not related to infection preventive-behavior (*b (SE)* = 0.02 (0.02)) in Oleksy et al. [26], whereas belief in government-related conspiracy narratives (*b (SE)* = − 0.04 (0.02)) were. Imhoff and Lamberty [32] found that belief in the conspiracy narrative that COVID-19 was a hoax had negative predictive power for pandemic-controlling behavior (S1: *b (SE)* = − 0.647 (0.109), *p* < 0.001; S2a: *b (SE)* = − 0.834 (0.092), *p* < 0.001; S2b: *b (SE)* = − 0.397 (0.109), *p* < 0.001), whereas belief that SARS-CoV-2 was human-made did not significantly negatively predict pandemic-controlling behavior (S1: *b (SE)* = 0.104 (0.104), *p* = 0.319; S2a: *b (SD)* = 0.104 (0.093), *p* = 0.265; S2b: *b (SD)* = 0.154 (0.082), *p* = 0.061). The largest negative association reported in the studies was between believing in a conspiracy narrative that views Jews as the authors of the virus and following the “stay home” directive: *OR* = 14.34, 95% CI [11.26, 18.25] [23]. Biddlestone et al. [33] concluded that conspiracy beliefs are negatively related to social distancing (*r* = − 0.16, *p* < 0.001; SEM model: *β* = − 0.04, *p* < 0.001) but not hygiene measures (*r* = − 0.02).

**Table 1:** Identified studies on infection-preventive behavior

References	Data collection	Sample	Surveyed variable(s)	Measures	Main findings
Allington et al. [30]	Study 1: 3.4–7.4 2020 Study 2: 1.4–3.4 2020 Study 3: 20.5–22.5 2020	Study 1: *N* = 949, *M*age (*SD*) = 36.35 (10.49); Study 2: *N* = 2250, *M*age (*SD*) = 45.47 (17.66); Study 3: *N* = 2254, *M*age (*SD*) = 43.93 (16.11); sample of UK-residents, Study 1: non-representative sample, Study 2 and 3: national representativeness with regard to age, gender, region, working status, social grade and education	Health-protective behavior	Study 1: 6 items on three conspiracy narratives on the origin of COVID-19 (true/false), 6 items on health-protective behaviors (yes/no); Study 2: 1 item on conspiracy belief, 5 items on health-protective behavior; Study 3: 5 items on conspiracy belief, 4 items on health-protective behavior	Study 1: Sig. negative relationship between conspiracy belief and health-protective behavior: *OR* = 0.46, 95% CI [0.34, 0.61], *p* < 0.001; Study 2: Sig. negative relationship between conspiracy belief and health-protective behavior: *OR* = 0.50, 95% CI [0.39, 0.66], *p* < 0.001; Study 3: Sig. negative relationship between conspiracy belief and health-protective behavior: *OR* = 0.37, 95% CI [0.29, 0.47], *p* < 0.001
Alper et al. [24]	Not reported	*N* = 1088, *M*age (*SD*) = 31.02 (39.43), non-representative Turkish sample	Preventive measures	Conspiracy belief: 2 items; preventive measures: 7 items, scale from 1 (strongly disagree) to 7 (strongly agree)	Correlation of conspiracy belief and preventive measures not significant: *r* = 0.02, *p* = 0.526
Biddlestone et al. [33]	4.4–13.4 2020	*N* = 704, *M*age (*SD*) = 37.26 (2.51), non-representative international sample (66 nationalities, mainly from the USA and UK)	Behaviors that reduce the spread of COVID-19	Conspiracy belief: 10 items, scale from 1 (strongly disagree) to 7 (strongly agree); 12 items, scale from 1 (definitely not) to 5 (definitely yes) behaviors that reduce the spread of COVID-19 (8 items social distancing intentions, 4 items hygiene intentions)	Negative, n. sig. association of hygiene intentions and conspiracy belief: *r* = − 0.02; negative sig. association of social distancing intentions and conspiracy belief: *r* = − 0.16*, p* < 0.001; conspiracy belief is a negative predictor of social distancing intentions in the SEM model: β = − 0.04, *p* < 0.001
Bierwiaczonek et al. [27]	16.3–20.4 2020	*N* = 403, *M*age (*SD*) = 37.42 (11.14), non-representative US American sample	Social distancing	Conspiracy belief: 3 items, scale from 1 (not at all) to 7 (very much) on three commonly shared conspiracy theories; 3 items, scale from 1 (strongly disagree) to 5 (strongly agree) on willingness to practice social distancing during the COVID-19 pandemic	Conspiracy belief predicts less social distancing at a later wave (T): Conspiracy belief T1 and social distancing T2: β = − 0.067, *p* = 0.002; Conspiracy belief T2 and social distancing T3: β = − 0.065, *p* = 0.002; Conspiracy belief T3 and social distancing T4: β = − 0.063, *p* = 0.002; Conspiracy belief T4 and social distancing T5: β = − 0.062, *p* = 0.002
Earnshaw et al. [28]	13.4–14.4 2020	*N* = 845, *M*age (*SD*) = 40.15 (11.67), non-representative US American sample	Complying with Public Health Recommendations	6-item questionnaire on different conspiracy narratives (agree/disagree); 8-item questionnaire on complying with public health recommendations (never, rarely, often, always)	No sig. association between complying with public health recommendations and conspiracy belief: *b* (*SE*) = − 0.04 (0.05), β = 0.2
Freeman et al. [23]	4.5–11.5 2020	*N* = 2501, *M*age (*SD*) = 46.6 (17.3), English sample, quota sampled to match the population for age, gender, income, and region	Following of UK government coronavirus guidance/future medical tests and treatments	Coronavirus conspiracy explanations: 48 items, scale from 1 (do not agree) to 5 (agree completely); Adherence to the government recommendations: 8 items, scale from 1 (not at all) to 5 (all of the time); Future medical tests and treatments: 6 items, scale from 1 (definitely) to 5 (definitely not)	Sig. negative association of conspiracy belief and adherence to guidelines/medical tests and treatments: *r* (− 0.27 to − 0.47), *p* < 0.001; Negative sig. association of conspiracy belief and adherence to guidelines: Conspiracy belief that COVID-19 is a bioweapon correlates negative with staying at home (*OR* = 4.57, 95% CI [3.62, 5.79]), endorsing the conspiracy narrative that Jews have created the virus to collapse the economy for financial gain was negatively associated with adhering to the guidance to stay at home (*OR* = 14.34, 95% CI [11.26, 18.25])
Garry et al. [31]	16.7–19.7 2020	*N* = 1045, representative sample of the English population in terms of gender, age, Government Office Region and 2019 vote	Adherence to current and future guidelines	6 items on different conspiracy narratives (strongly disagree, disagree, slightly disagree, slightly agree, agree, strongly agree, don’t know); 3 items on adherence to current guidelines, scale from 1 (not at all) to 7 (completely), 5 items on adherence to future guidelines (yes definitely, yes probably, no probably not, no definitely not, don’t know)	Conspiracy belief predicts current and future non-adherence significantly: *b* (*SE*) = 0.32 (0.03), β = 0.36, *p* < 0.001; future: *b* (SE) = 0.35 (0.03), β = 0.38, *p* < 0.001
Imhoff and Lamberty [32]	20.3–25.3 2020	Study 1: *N* = 220, *M*age (*SD*) = 40.18 (12.33), US American sample; Study 2a: *N* = 288, *M*age (*SD*) = 36.60 (11.16), US American sample*;*Study 2b: *N* = 298, *M*age (*SD*) = 37.29 (12.79), sample from UK; non-representative samples	Containment-related behavior	Conspiracy belief: two sets (COVID-19 hoax and SARS-CoV-2 human-made) with 3 items each, scale from 1 (strongly disagree) to 7 (strongly agree); 7 items on containment-related behavior, scale from 1 (never) to 7 (always/strongly)	Study 1: Sig. negative association between conspiracy belief COVID-19 is a hoax and containment-related behavior: *r* = − 0.356, *p* < 0.002; *b* (*SE*) = − 0.647 (0.109), *p* < 0.001; Association conspiracy belief SARS-CoV-2 human-made and containment-related behavior n. sig. *r* = − 0.123 (n. sig.); *b* (*SE*) = 0.104 (0.104), *p* = 0.319; Study 2: Negative association between COVID-19 hoax and containment-related behavior: Study 2a: *r* = − 0.524 (sig.), *b* (*SE*) = − 0.834 (0.092), *p* < 0.001; Study 2b: *r* = − 0.154 (n. sig.), *b* (*SE*) = − 0.397 (0.109), *p* < 0.001); Association SARS-CoV-2 human-made: Study 2a: *r* = − 0.307 (sig., *p* < 0.001); *b* (*SE*) = 0.104 (0.093), *p* = 0.265; Study 2b: *r* = 0.014 (n. sig.), *b* (*SE*) = 0.154 (0.082), *p* = 0.061)
Kowalski et al. [25]	11.4–14.4 2020; 21.4–28.4 2020	Study 1: *N* = 507, *M*age (*SD*) = 44.07 (14.41), representative Polish sample in terms of gender and settlement size; Study 2: *N* = 840, *M*age (*SD*) = 29.94 (10.39), non-representative Polish sample	Adherence to safety guidelines	14-item (Study 1) and 12-item questionnaire (Study 2) on different conspiracy narratives, scales from 1 (strongly disagreeing) to 7 (strongly agreeing); 4-item (Study 1) and 5-item questionnaire (Study 2) on adherence to safety and self-isolation guidelines, 7-level scale	Sig. negative association between conspiracy belief and adherence to safety guidelines: *rs* = − 0.22; conspiracy belief predicted adherence to safety guidelines (sig.): Study 1: *b* (*SE*) = − 0.05 (0.01), *p* ≤ 0.001; Study 2: *b* (*SE*) = − 0.08 (0.01), *p* < 0.001
Oleksy et al. [26]	13.3–15.3 2020	Study 1: *N* = 1046, representative Polish sample in terms of gender, age and size of residence	Protective behavior	Conspiracy belief: 3 items on general conspiracy narratives, 2 items on government-related conspiracy narratives; Protective behavior: 9 items (yes/no)	Government-related and general conspiracy belief correlated n. sig. with protective behavior: *r* = 0.01 and *r* = 0.05; Government-related conspiracy belief sig. negatively predicted protective behavior: *b* (*SE*) = − 0.04 (0.02); General conspiracy belief n. sig.: *b* (*SE*) = 0.02 (0.02)
Romer and Jamieson [29]	17.3–27.3 2020; 10.7–21.7 2020	*N* = 840, representative US American sample in terms of age, gender, race/ethnicity and education	Taking of preventive actions	Conspiracy belief: 3-item questionnaire (3 different conspiracy narratives), scale from 1 (definitely false) to 4 (definitely true); Preventive actions: list of 9 actions to prevent further spread of COVID-19 (yes/no)	Conspiracy belief in March sig. predicted actions taken in July: β = −0.16, 99% CI [− 0.26, − 0.07], *p* ± 0.005
Teovanović et al. [22]	10.4–22.4 2020	*N* = 407, *M*age (*SD*) = 34.88 (12.81), non-representative Serbian sample	Adherence to COVID-19 guidelines by the WHO and the Serbian Ministry of Health	13 items on conspiracy belief, scale from 1 (completely disagree) to 5 (completely agree); 12 items on adherence to guidelines, thereof 5 items on preventive behavior on a scale from 1 (never) to 5 (very often), 7 items on avoided risk behaviors (never, once, twice, three times, > three times)	Conspiracy belief sig. negatively correlated with the adherence to COVID-19 guidelines: *r* = −0.17 *p* < 0.05; Conspiracy belief was a sig. negative predictor for adherence to COVID-19-guidelines: *b* (*SE*) = − 0.09 (0.03), 95% CI [− 0.14, − 0.04], *p* = 0.001

Sig. = significant, n.sig. = not significant

The total sample size of the ten studies that examined vaccination willingness is *N* = 20,210. The smallest sample counts *N* = 396 participants [34], the largest sample *N* = 5114 participants [35], and the average age ranges from 26 years [34] to 47 years [35]. Two studies tested individuals from the USA [28, 29] three from the UK [23, 31, 35], and one sample each is from Serbia [22], Italy [36], and France [34]. In addition, there is a study with samples from both Turkey and the UK [37] and an Arabic sample with participants from 18 nations, with the majority coming from Jordan and Kuwait [38]. Conspiracy beliefs were measured in different ways. Prati [36] and Salali and Uysal [37] each used one item that asked about conspiracy belief in the non-natural origin of COVID-19, while Freeman, Waite, et al. [23] used 48 items to cover belief in a wide variety of conspiracy narratives. Bertin et al. [34] distinguished three groups of conspiracy narratives that addressed supposed machinations of different groups: a threatening foreign outgroup (China), unspecified outgroups (e.g., industrialists), and a national ingroup (French government). Vaccination willingness, or vaccination hesitancy [35], was assessed with one item each. Studies reported either correlation coefficients [22, 23, 34, 35], odds ratios [23, 28, 36–38], standardized regression coefficients [29, 34], or unstandardized regression coefficients [22, 28, 31]. Freeman and Loe et al. [35] constructed a SEM model. Nine out of ten of the reviewed studies demonstrated a negative correlation of conspiracy belief and vaccination willingness. The smallest reported correlation was − 0.23 [34], and the largest was − 0.53 [22]. The various regression analyses yielded odds ratios ranging from 0.26 [28] to 2.73 [38], standardized regression coefficients from − 0.11 [34] to 0.35 [31], and unstandardized regression coefficients from 0.45 [31] to 1.63 [35]. In the SEM model of Freeman, Loe, et al. [35], conspiracy belief and vaccine hesitancy were positively associated: *β* = 0.38, *p* < 0.001. Prati [36] concluded in her study that the conspiracy belief in non-natural origins was not a significant negative predictor of willingness to vaccinate: *OR* = 0.88, 95% CI [0.22, 3.55]. There is a difference in the strength of the association depending on the content of the conspiracy narratives underlying the conspiracy belief variable. For example, the conspiracy belief in the artificial origin of COVID-19 was not significantly correlated with vaccination willingness in Prati [36]. Also in Salali and Uysal [37], UK: *OR* = 0.78, 95% CI [0.49, 1.25], *p* = 0.31; Turkey: *OR* = 0.65, 95% CI [0.53, 0.79], *p* < 0.001, and in Sallam et al. [38], *OR* = 0.47, CI [0.38, 0.57], the correlations were rather small. In contrast, conspiracy beliefs that COVID-19 vaccination would be used for microchip implantation, *OR* = 2.39, 95% CI [1.72, 3.30], or that vaccination would cause infertility, *OR* = 2.73, 95% CI [1.90, 3.93], were highly positive associated with vaccine hesitancy [38].

**Table 2:** Identified studies on vaccination willingness

Authors, publication date	Data collection	Sample	Surveyed variable	Measures	Main findings
Bertin et al. [34]	17.4–25.4 2020	*N* = 396, *M*_age_ (*SD*) = 26.1 (10.3), non-representative French sample	Vaccination willingness	Conspiracy belief: Scales from 1 (strongly disagree) to 5 (strongly agree), 7 items on Outgroup-conspiracies, 3 items on Ingroup-conspiracies; Vaccination willingness: 1 item, scale from 1 (I would definitely not be vaccinated under any circumstances) to 7 (I would be vaccinated without hesitation)	Conspiracy belief sig. negatively correlated with vaccination willingness: Outgroup: *r* = − 0.23, *p* < 0.001; Ingroup: *r* = − 0.28, *p* < 0.001; Conspiracy belief is a sig. negative predictor for vaccination willingness: Outgroup: β = − 0.23, 95% CI [− 0.34, − 0.12], *p* < 0.001; Ingroup: β = − 0.11, 95% CI [− 0.22, − 0.01], *p* = 0.05
Earnshaw et al. [28]	13.4–14.4 2020	*N* = 845, *M*_age_ (*SD*) = 40.15 (11.67), non-representative US American sample	Vaccination willingness	Conspiracy belief: 6-item questionnaire on different conspiracy narratives (agree/disagree); Vaccination willingness: 1 item on a 5-level Likert-scale	72.9% of the participants believing in conspiracies were willing to get vaccinated vs. 92.0% of those who don’t believe in conspiracies, X^2^ (1) = 55.72, *p* ≤ 0.01; Sig. negative association of vaccination willingness and conspiracy belief: *b* (*SD*) = − 1.37 (0.28), *OR* = 0.26, 95% CI [0.15–0.44], *p* ≤ 0.01
Freeman et al. [35]	24.9–17.10 2020	*N* = 5114, *M*_age_ (*SD*) = 46.9 (17.1), representative sample from the UK regarding age, gender, ethnicity, income and region	Vaccination hesitancy	OCEANS coronavirus conspiracy scale: 14 items on conspiracy belief, scale from 1 (do not agree) to 5 (agree completely); Oxford COVID-19 hesitancy scale: 7 items on vaccination hesitancy, scale from 1 to 5 (higher score equals higher vaccination hesitancy)	Conspiracy belief correlated with vaccination hesitancy: *r* = 0.38, *p* < 0.001; Conspiracy belief sig. predicted vaccination hesitancy: *b* (*SD*) = 1.63 (0.05), *p* < 0.001; SEM model: β = 0.39, *p* < 0.001
Freeman et al. [23]	4.5–11.5 2020	*N* = 2501, *M*_age_ (*SD*) = 46.6 (17.3), English sample, quota sampled to match the population for age, gender, income, and region	Willingness to take diagnostic or antibody tests or to be vaccinated	Conspiracy belief: 48 items, scale from 1 (do not agree) to 5 (agree completely); Future medical tests and treatment: inc. 1 item on vaccination willingness: 6 items, scale from 1 (definitely) to 5 (definitely not)	Sig. negative correlation of specific and general conspiracy belief and vaccination willingness: *r* = 0.35 and *r* = 0.37, *p* < 0.001; Participants that believed COVID-19 is a bioweapon (*OR* = 2.11, 95% CI [1.65, 2.70]) or Jews created the virus for financial gain (*OR* = 2.70, 95% CI [2.08, 3.50]) reported less vaccination willingness then people not holding conspiracy beliefs
Garry et al. [31]	16.7–19.7 2020	*N* = 1045, representative sample of the English population	Vaccination willingness	6 items on different conspiracy narratives on a 7-level scale (strongly disagree, disagree, slightly disagree, slightly agree, agree, strongly agree, don’t know); 1 item on vaccination willingness on a 5-level scale (yes definitely, yes probably, no probably not, no definitely not, don’t know)	Conspiracy belief sig. negatively predicted vaccination unwillingness: *b* (*SD*) = 0.45 (0.05), β = 0.35, *p* < 0.001
Prati [36]	April 2020	*N* = 624, *M*_age_ (*SD*) = 32.31 (12.69), non-representative Italian sample	Vaccination willingness	Conspiracy belief: 1 item (yes/no/don’t know); 1 item on vaccination willingness (yes/no/don’t know)	Conspiracy belief was no sig. predictor for vaccination unwillingness: *OR* = 0.88, 95% CI [0.22, 3.55]
Romer and Jamieson [29]	17.3–27.3 2020; 10.7–21.7 2020	*N* = 840, representative US American sample in terms of age, gender, race/ethnicity and education	Vaccination willingness	Conspiracy belief: 3-item questionnaire (3 different conspiracy narratives), scale from 1 (definitely false) to 4 (definitely true) 1 item on vaccination willingness scale from 1 (not at all likely) to 4 (very likely)	Conspiracy belief in March negatively predicted vaccination willingness in July: β = − 0.29, 99% CI [− 0.37, − 0.22], *p* ± 0.005
Salali and Uysal [37]	May 2020	UK: *N* = 1088, *M*_age_ (*SD*) = 31.92 (11.2), Turkey: *N* = 3936, *M*_age_ (*SD*) = 44.33 (13.70), non-representative samples	Vaccination willingness	Conspiracy belief: 1 item (natural/artificial/not sure); 1 item on vaccination willingness (yes/no/not sure)	Conspiracy belief about the artificial origin of the virus was associated with sig. less vaccination willingness, UK: artificial origin *OR* = 0.78, 95% CI [0.49, 1.25], *p* = 0.31; natural origin, *OR* = 2.63, 95% CI [1.83, 3.78], *p* < 0.001; Turkey: artificial origin *OR* = 0.65, 95% CI [0.53, 0.79], *p* < 0.001; natural origin, *OR* = 2.26, 95% CI [1.93, 2.66], *p* < 0.001
Sallam et al. [38]	14.12–18.12 2020	*N* = 3414, non-representative Arabic sample (mostly from Jordan (63.6%) and Kuwait (22.6%), plus 16 other Arab states)	Vaccination willingness	4 items on conspiracy narratives (yes/no, resp. natural source/man-made virus), 1 item on vaccination willingness (yes/no)	Conspiracy belief was associated with sig. less vaccination willingness (95% CI, *p* < 0.001): COVID-19 man-made: *OR* = 0.47, CI [0.38, 0.57]; COVID-19 was manufactured to force the public to get vaccinated: *OR* = 1.89, CI [1.46, 2.43]; COVID-19 vaccine is used to implant microchips into people to control them: *OR* = 2.39, CI [1.72, 3.30]; COVID-19 vaccine will lead to infertility: *OR* = 2.73, CI [1.90, 3.93]
Teovanović et al. [22]	10.4–22.4 2020	*N* = 407, *M*_age_ (*SD*) = 34.88 (12.81), non-representative Serbian sample	Vaccination willingness	13 items on conspiracy narratives, scale from 1 (completely disagree) to 5 (completely agree); 1 item on vaccination willingness, scale from 1 (definitely would not) to 5 (definitely would)	Conspiracy belief correlates sig. negatively with vaccination willingness: *r* = − 0.53, *p* < 0.05; Conspiracy belief sig. negative predictor for vaccination willingness: *b* (*SD*) = − 0.92 (0.07), 95% CI [− 1.07, − 0.78], *p* < 0.002

Sig. = significant, n.sig. = not significant

## Discussion

The systematic literature review on the link between COVID-19-related conspiracy belief and infection-preventive behavior and vaccination willingness presented here provides clear evidence in favor of a negative association between conspiracy belief and infection-preventive behavior and vaccination willingness. Ten of twelve of the reviewed studies demonstrated a negative association of COVID-19-related conspiracy beliefs with infection-preventive behavior, and nine of ten studies showed a negative association with vaccination willingness. Studies on vaccination willingness showed a largely consistent picture. The reported correlation coefficients showed a medium to high linear relationship of the variables, with values ranging from − 0.23 [34] to − 0.53 [22]. While simple correlations typically have a high face validity and can be straightforwardly interpreted also by laypersons, this may sometimes not do justice to the complexity of the subject matter, because they do not allow to control for other predictors, unlike regression coefficients. Another advantage of regression coefficients is that regression coefficients are robust even with small sample sizes. The various regression analyses demonstrated the negative relationship between conspiracy belief and willingness to get vaccinated, largely with small to very small effect sizes, and in some cases with a medium effect [39]. Only Prati [36] found no negative association between COVID-19-related conspiracy beliefs and vaccination willingness. This could be related to the fact that only a single item asking about the non-natural origin of COVID-19 was used. This is also true for the study by Salali and Uysal [37], who also asked about the belief in the non-natural origin of COVID-19 and demonstrated a negative association between conspiracy belief and vaccination willingness. One explanation for the differences in effect sizes could be that the different conspiracy narratives, and thus the content of each conspiracy belief, is crucial for the strength of the association. Not only Prati [36] and Salali and Uysal [37] looked at the belief in the artificial origin of COVID-19. Also Sallam et al. [38] found an association: *OR* = 0.47, 95% CI [0.38, 0.57] but it was smaller than the relationship between the conspiracy belief that COVID-19 vaccination would be used for microchip implantation (*OR* = 2.39, 95% CI [1.72, 3.30]) or that vaccination would cause infertility (*OR* = 2.73, 95% CI [1.90, 3.93]) and vaccine hesitancy [38]. This could indicate a mediating factor, such as threat- or risk perception that moderates the association between conspiracy beliefs and vaccination willingness, and that varies in intensity depending on the narrative. One might assume that a person who believes that the virus was fabricated in a laboratory would nevertheless believe it to be dangerous and be willing to protect themself with a vaccine. However, if the vaccine itself is integral part of the conspiracy belief and is therefore thought to be more dangerous than the disease it protects against, low willingness to vaccinate is not surprising [22]. Here, not only risk or threat perception, but also the resulting COVID-19-related anxiety and the associated degree to which individuals perceive themselves as affected by the conspiracy could mediate the association with infection-preventing behavior, which would be consistent with the findings of the Brewer et al. [40] meta-analysis. An indirect influence of conspiracy beliefs via feelings of powerlessness, as addressed by Jolley and Douglas [13, 15] in their work, which could lead to reduction in infection-preventive behavior or vaccination willingness, is also possible. Moreover, the present data suggest that conspiracy beliefs of any kind are negatively related to vaccination willingness. This could be explained by a core motive of conspiracy belief: People who believe in conspiracy narratives assume that important information, such as the truth about the harmfulness of vaccines or the origin of the pandemic, is being hidden from the public [41, 42]. This may explain why they are suspicious of vaccination campaigns by the state and of vaccination itself, even if they believe the virus is dangerous. Discouragement of vaccination might as well be mediated through social media usage patterns. Chadwick et al. demonstrated that people with a high conspiracy mentality who primarily seek information on social media platforms discourage vaccination [43]. Harmful misinformation spread on social media and the risk of drifting into ideologically isolated echo chambers could therefore contribute to the perpetuation of the pandemic situation. A challenge also recognized by the WHO, who stated we are not only fighting a pandemic but also an infodemic [21]. Especially in the fast-paced pandemic situation, analysis of vaccine-related content on social media provides important information on public opinion [44].

The varying strength of the association with infection-preventive behavior could also be explained in terms of the content of the conspiracy narratives studied. In Imhoff and Lamberty [32], for example, individuals who believed that the pandemic was a hoax perceived the pandemic as less threatening, whereas there was no significant relationship between believing that the virus was human-made and threat perception. Belief in the conspiracy narrative that COVID-19 is a hoax was negatively related to infection-preventive behavior, whereas the relation with the belief that SARS-CoV-2 is human-made was less clear. In both US-samples the negative correlation between believing COVID-19 is a hoax and infection-preventive behavior was significant, in the UK it was not. The correlations between believing SARS-CoV-2 is human-made varied in both countries regarding their direction and significance. These findings suggest that a conspiracy, which pictures the pandemic as less threatening, may lead to reduced adherence to preventive behavior. While Oleksy et al. [26] found no significant association between general COVID-19-related conspiracy beliefs and infection-preventive behavior, there was a negative association with beliefs in government-related conspiracy narratives. The population is most prominently urged by the government to engage in protective behavior, and the government imposes infection prevention measures to this end. The suggestion is that conspiracy narratives about hidden malicious intent or government misconduct therefore reduce infection-preventive behavior among the population. However, it must be critically noted here that Oleksy et al. only found a small difference between the two sets of conspiracy narratives, which could possibly also have been caused by a differential measurement error. Also noteworthy is the largest negative association reported in the studies: Belief in the conspiracy narrative that Jews created the virus to collapse the economy and benefit financially from was strongly negative related to stay-at-home behavior in a UK sample: *OR* = 14.34, 95% CI [11.26, 18.25] [23]. According to a study by Staetsky [45] 13% of the population in the UK holds antisemitic prejudices which could be an explanation for this surprisingly large association. COVID-19-related conspiracy narratives may help to satisfy the need to make sense of events during a period, which is characterized by uncertainty, thereby regaining a sense of control [46, 47]. Another explanation for why conspiracy beliefs and infection-preventive behavior are negatively connected is addressed by Bierwiaczonek et al. [27] who argue that belief in an external threat may lead people to seek social support, and thus prevent the minimization of social contact in the interest of infection prevention. This could also explain the findings of Biddlestone et al. [33], who found that conspiracy beliefs are negatively related to social distancing (*r* = − 0.16, *p* < 0.001; SEM model: *β* = − 0.04, *p* < 0.001) but not hygiene measures (*r* = − 0.02). Alper et al. [24] and Earnshaw et al. [28] found no significant association between conspiracy beliefs and infection-preventive behavior. However, in Alper et al. [24], the reliability of the measurement of conspiracy beliefs is questionable, as only a rather general item (“Coronavirus was developed and spread around the world by certain people for their own purposes”) and an inversely coded version of the item were used to capture the construct. Earnshaw et al. [28] used a 6-item questionnaire, arguably a more reliable measurement tool, but again, as in most of the studies reviewed, the reliability and validity of the measurements are not assessed. This is not surprising, because due to the novelty of the studied construct, no established instruments were readily available. As a consequence, most of the authors of the reviewed studies decided to develop their own instruments, for example to assess infection-preventive behavior in the context of the COVID-19 pandemic. In many of the studies reviewed, the selection of the surveyed preventive behavior was based on either government guidelines or those of the WHO. Other authors developed questionnaires based on studies of other infectious diseases, such as HIV [28]. For those instruments that were adapted, it is unclear to which extent the reliability and validity of the measurements can be applied to the novel context of the COVID-19 pandemic. There is a broad consensus that it is of great importance to check the consistency of results across time or across different observers (i.e., the issue of reliability) and to carefully check whether the instruments used actually measure what they are supposed to measure (i.e., the issue of validity). However, these comprehensive quality controls come at the expense of the rapid dissemination and implementation of the findings, which has to be weighed carefully. Evidently, this issue is not limited to highly dynamic situations like a pandemic. In their systematic review, Goreis and Voracek [10] found substantial heterogeneity in the operationalization and hence a lack of scientific consensus in psychological research on conspiracy narratives in general. When interpreting the present findings, the aspect of the consistency and the accuracy of the measurement instruments should therefore always be taken into account. A further aspect that may be relevant is the heterogeneity in the samples studied and their thus highly varying representativeness (see Tables 1, 2). A limitation of the present work are the broad definitions of the variables COVID-19-related conspiracy belief and infection-preventive behavior. The inclusion criteria could have been further specified to reliably identify more specific effects of conspiracy narratives on particular behavior. Another limitation is the measure of vaccination willingness. It remains unclear to what extent expressed intention to be vaccinated is related to actual behavior. Since the literature search was conducted, other relevant papers have been published (e.g., [47–49]), which is not surprising given the dynamic nature and the global relevance of the pandemic. This review focuses on the literature published during the first year of the pandemic. Thus, developments that occurred during the second pandemic year such as broadly implemented government vaccination campaigns, media coverage on the effectiveness and side effects of vaccination, and the discourse on restricted social participation for unvaccinated people, are not reflected in it. The present review only summarizes the state of the literature and knowledge at the particular point in time at which the literature search was conducted, which represents a limitation. However, it may also serve as an important reference point to track the evolution of COVID-19-related conspiracy belief over time (e.g., after the second year of the pandemic).

## Conclusion

Belief in COVID-19-related conspiracy narratives is negatively associated with vaccination willingness and infection-preventive behavior. These findings are consistent with previous research linking conspiracies and health-related preventive behavior and suggest that the content of conspiracy narratives is associated with the strength of the effect. Even very small negative effects on taking behavior to contain the pandemic translate into higher rates of infection, thus affecting the health of countless people long-term and resulting in a cost in human lives. Reduced vaccination willingness due to belief in conspiracies could significantly impede successful control of the pandemic, thus prolonging the current crisis. Because belief in COVID-19-related conspiracy narratives and resistance to preventive behavior and future vaccination are closely related, it could be critical to counter them to contain the spread of the virus. Key to successful combating is a better understanding of the phenomenon of conspiracy narrative. Further research projects should focus on the question of what specific conspiracy narratives, or what types of conspiracy narratives, are related to which specific behavior resulting in pandemic progression and ask what variables moderate this effect. Also, research on the question of how conspiracy narratives spread is advisable. Furthermore, a review of the published literature after the second year of the pandemic would be helpful to assess to what extend COVID-19-related conspiracy belief is influenced by factors such as new findings on vaccination or restrictions for unvaccinated individuals and the public debate about it. Moreover, a precise definition and consistent, psychological operationalization of the construct conspiracy belief with differentiated measurement instruments would be beneficial to increase the quality of the results and to ensure their comparability. Systematic reviews have the potential to generate insights that go beyond those of a single study, because studies differ in many different aspects such as the time and place of the data collection and sample characteristics, to name but a few. Especially in a dynamic and complex situation such as a pandemic, systematic reviews are needed to provide more people with a structured overview of scientifically sound information.

## Supplementary Information

**Additional file 1.** Dataset.

## Data Availability

The dataset supporting the conclusions of this article is available in the OSF repository, at https://osf.io/6cgds/?view_only=1efe8be1cc384e2689829201e52b3418.

## References

[1] van Prooijen JW, Douglas KM (2017). Conspiracy theories as part of history: the role of societal crisis situations. Mem Stud.

[2] LeBoeuf RA, Norton MI (2012). Consequence-cause matching: looking to the consequences of events to infer their causes. J Consum Res.

[3] Leman P, Cinnirella M (2007). A major event has a major cause: evidence for the role of heuristics in reasoning about conspiracy theories. Soc Psychol Rev.

[4] Spinney L (2017). Pale rider: the Spanish flu of 1918 and how it changed the World.

[5] Bangerter A, Krings F, Mouton A, Gilles I, Green EGT, Clémence A (2012). Longitudinal investigation of public trust in institutions relative to the 2009 H1N1 pandemic in Switzerland. PLoS ONE.

[6] Sanche S, Lin YT, Xu C, Romero-Severson E, Hengartner N, Ke R (2020). High contagiousness and rapid spread of severe acute respiratory syndrome coronavirus 2. Emerg Infect Dis.

[7] Salmon DA, Dudley MZ, Brewer J, Kan L, Gerber JE, Budigan H (2021). COVID-19 vaccination attitudes, values and intentions among United States adults prior to emergency use authorization. Vaccine.

[8] Earnshaw VA, Bogart LM, Klompas M, Katz IT (2019). Medical mistrust in the context of Ebola: implications for intended care-seeking and quarantine policy support in the United States. J Health Psychol.

[9] Gillman J, Davila J, Sansgiry S, Parkinson-Windross D, Miertschin N, Mitts B (2013). The effect of conspiracy beliefs and trust on HIV diagnosis, linkage, and retention in young MSM with HIV. J Health Care Poor Underserved.

[10] Goreis A, Voracek M (2019). A systematic review and meta-analysis of psychological research on conspiracy beliefs: field characteristics, measurement instruments, and associations with personality traits. Front Psychol.

[11] Stanley M, Barr N, Peters K, Seli P. Analytic-thinking predicts hoax beliefs and helping behaviors in response to the COVID-19 Pandemic2020.

[12] Oliver JE, Wood T (2014). Medical conspiracy theories and health behaviors in the United States. JAMA Intern Med.

[13] Jolley D, Douglas K (2014). The social consequences of conspiracism: exposure to conspiracy theories decreases intentions to engage in politics and to reduce one’s carbon footprint. Br J Psychol.

[14] Hornsey MJ, Harris EA, Fielding KS (2018). The psychological roots of anti-vaccination attitudes: a 24-nation investigation. Health Psychol.

[15] Jolley D, Douglas K (2014). The effects of anti-vaccine conspiracy theories on vaccination intentions. PLoS ONE.

[16] Greenwood B (2014). The contribution of vaccination to global health: past, present and future. Philos Trans R Soc Lond B Biol Sci.

[17] Hodgson SH, Mansatta K, Mallett G, Harris V, Emary KRW, Pollard AJ (2021). What defines an efficacious COVID-19 vaccine? A review of the challenges assessing the clinical efficacy of vaccines against SARS-CoV-2. Lancet Infect Dis.

[18] Lu D, Aleta A, Ajelli M, Pastor-Satorras R, Vespignani A, Moreno Y. Data-driven estimate of SARS-CoV-2 herd immunity threshold in populations with individual contact pattern variations2021.

[19] Kenyon C (2020). Flattening-the-curve associated with reduced COVID-19 case fatality rates—an ecological analysis of 65 countries. J Infect.

[20] Douglas KM, Uscinski JE, Sutton RM, Cichocka A, Nefes T, Ang CS (2019). Understanding conspiracy theories. Polit Psychol.

[21] WHO. Rolling updates on coronavirus disease (COVID-19). 2021. https://www.who.int/emergencies/diseases/novel-coronavirus-2019/events-as-they-happen.

[22] Teovanović P, Lukić P, Zupan Z, Lazić A, Ninković M, Žeželj I (2020). Irrational beliefs differentially predict adherence to guidelines and pseudoscientific practices during the COVID-19 pandemic. Appl Cogn Psychol.

[23] Freeman D, Waite F, Rosebrock L, Petit A, Causier C, East A (2020). Coronavirus conspiracy beliefs, mistrust, and compliance with government guidelines in England. Psychol Med.

[24] Alper S, Bayrak F, Yilmaz O (2020). Psychological correlates of COVID-19 conspiracy beliefs and preventive measures: evidence from Turkey. Curr Psychol.

[25] Kowalski J, Marchlewska M, Molenda Z, Górska P, Gawęda Ł (2020). Adherence to safety and self-isolation guidelines, conspiracy and paranoia-like beliefs during COVID-19 pandemic in Poland—associations and moderators. Psychiatry Res.

[26] Oleksy T, Wnuk A, Maison D, Łyś A (2021). Content matters. Different predictors and social consequences of general and government-related conspiracy theories on COVID-19. Pers Individ Dif.

[27] Bierwiaczonek K, Kunst JR, Pich O (2020). Belief in COVID-19 conspiracy theories reduces social distancing over time. Appl Psychol Health Well Being.

[28] Earnshaw VA, Eaton LA, Kalichman SC, Brousseau NM, Hill EC, Fox AB (2020). COVID-19 conspiracy beliefs, health behaviors, and policy support. Transl Behav Med.

[29] Romer D, Jamieson KH (2020). Conspiracy theories as barriers to controlling the spread of COVID-19 in the U.S.. Soc Sci Med.

[30] Allington D, Duffy B, Wessely S, Dhavan N, Rubin J (2020). Health-protective behaviour, social media usage and conspiracy belief during the COVID-19 public health emergency. Psychol Med.

[31] Garry J, Ford R, Johns R (2020). Coronavirus conspiracy beliefs, mistrust, and compliance: taking measurement seriously. Psychol Med.

[32] Imhoff R, Lamberty P (2020). A bioweapon or a hoax? The link between distinct conspiracy beliefs about the coronavirus disease (COVID-19) outbreak and pandemic behavior. Soc Psychol Personal Sci.

[33] Biddlestone M, Green R, Douglas KM (2020). Cultural orientation, power, belief in conspiracy theories, and intentions to reduce the spread of COVID-19. Br J Soc Psychol.

[34] Bertin P, Nera K, Delouvée S (2020). Conspiracy Beliefs, rejection of vaccination, and support for hydroxychloroquine: a conceptual replication-extension in the COVID-19 pandemic context. Front Psychol.

[35] Freeman D, Loe BS, Chadwick A, Vaccari C, Waite F, Rosebrock L (2020). COVID-19 vaccine hesitancy in the UK: the Oxford coronavirus explanations, attitudes, and narratives survey (Oceans) II. Psychol Med.

[36] Prati G (2020). Intention to receive a vaccine against SARS-CoV-2 in Italy and its association with trust, worry and beliefs about the origin of the virus. Health Educ Res.

[37] Salali GD, Uysal MS (2020). COVID-19 vaccine hesitancy is associated with beliefs on the origin of the novel coronavirus in the UK and Turkey. Psychol Med.

[38] Sallam M, Dababseh D, Eid H, Al-Mahzoum K, Al-Haidar A, Taim D (2021). High rates of COVID-19 vaccine hesitancy and its association with conspiracy beliefs: a study in Jordan and Kuwait among other Arab countries. Vaccines.

[39] Rosenthal JA (1996). Qualitative descriptors of strength of association and effect size. J Soc Serv Res.

[40] Brewer NT, Chapman GB, Gibbons FX, Gerrard M, McCaul KD, Weinstein ND (2007). Meta-analysis of the relationship between risk perception and health behavior: the example of vaccination. Health Psychol.

[41] Lukić P, Žeželj I, Stanković B (2019). How (ir)rational is it to believe in contradictory conspiracy theories?. Eur J Psychol.

[42] Wood MJ, Douglas KM, Sutton RM (2012). Dead and alive: beliefs in contradictory conspiracy theories. Soc Psychol Personal Sci.

[43] Chadwick A, Kaiser J, Vaccari C, Freeman D, Lambe S, Loe BS (2021). Online social endorsement and Covid-19 vaccine hesitancy in the United Kingdom. Soc Media Soc..

[44] Eibensteiner F, Ritschl V, Nawaz FA, Fazel SS, Tsagkaris C, Kulnik ST (2021). People’s willingness to vaccinate against COVID-19 despite their safety concerns: Twitter poll analysis. J Med Internet Res.

[45] Staetsky D (2017). Antisemitism in contemporary Great Britain: a study of attitudes towards Jews and Israel.

[46] Douglas KM, Sutton RM, Cichocka A (2017). The psychology of conspiracy theories. Curr Dir Psychol Sci.

[47] van Prooijen JW, van Dijk E (2014). When consequence size predicts belief in conspiracy theories: the moderating role of perspective taking. J Exp Soc Psychol.

[48] Maftei A, Holman AC. SARS-CoV-2 Threat Perception and Willingness to Vaccinate: The Mediating Role of Conspiracy Beliefs. Front Psychol. 2021;12(3371). 10.3389/fpsyg.2021.672634 PMC8417247 34489791

[49] Islam MS, Kamal A-HM, Kabir A, Southern DL, Khan SH, Hasan SMM (2021). COVID-19 vaccine rumors and conspiracy theories: The need for cognitive inoculation against misinformation to improve vaccine adherence. PLOS ONE..

