# Supplementary material for: Systematic review on the association of COVID-19-related conspiracy belief with infection-preventive behavior and vaccination willingness
Source: BMC Psychol. 2022 Mar 15;10:66. doi: 10.1186/s40359-022-00771-2 (PMC8923094; doi:10.1186/s40359-022-00771-2)
Supplement: Supplementary file 1 — Additional file 1. Dataset. [file 40359_2022_771_MOESM1_ESM.docx]

# Appendix

**Table A1**
*Documentation of the systematic literature review*

| Database | Term | Results |
| --- | --- | --- |
| COVID-19 Data Portal | (conspiracy theories OR conspiracy beliefs) AND (COVID-19 OR coronavirus) | 54 |
| APA PsycArticles | (conspiracy theories OR conspiracy beliefs) AND (COVID-19 OR coronavirus) | 11 |
| **Psychology and Behavioral Sciences** | (conspiracy theories OR conspiracy beliefs) AND (COVID-19 OR coronavirus) | 5 |
| Scopus | (conspiracy theories OR conspiracy beliefs) AND (COVID-19 OR coronavirus) | 22 |
| PubMed | (conspiracy theories OR conspiracy beliefs) AND (COVID-19 OR coronavirus) | 47 |
| Total |  | 139 |

**Table A2**
*Included studies*

| Authors, Publication | Title | Surveyed variable(s) |
| --- | --- | --- |
| Allington et al., 2020 | Health-protective behavior, social media usage and conspiracy belief during the COVID-19 public health emergency | IPB |
| Alper et al, 2020 | Psychological correlates of COVID-19 conspiracy beliefs and preventive measures: Evidence from Turkey | IPB |
| Bertin et al., 2020 | Conspiracy Beliefs, Rejection of Vaccination, and Support for hydroxychloroquine: A Conceptual Replication-Extension in the COVID-19 Pandemic Context | VW |
| Biddlestone et al., 2020 | Cultural orientation, power, belief in conspiracy theories, and intentions to reduce the spread of COVID‐19 | IPB |
| Bierwiaczonek et al., 2020 | Belief in COVID-19 Conspiracy Theories Reduces Social Distancing over Time | IPB |
| Earnshaw et al., 2020 | COVID-19 conspiracy beliefs, health behavior, and policy support | IPB & VW |
| Freeman, Waite et al., 2020 | Coronavirus conspiracy beliefs, mistrust, and compliance with government guidelines in England | IPB & VW |
| Freeman, Loe et al., 2020 | COVID-19 vaccine hesitancy in the UK: the Oxford coronavirus explanations, attitudes, and narratives survey (Oceans) II | VW |
| Garry et al., 2020 | Coronavirus conspiracy beliefs, mistrust, and compliance: taking measurement seriously | IPB & VW |
| Imhoff & Lamberty, 2020 | A Bioweapon or a Hoax? The Link Between Distinct Conspiracy Beliefs About the Coronavirus Disease (COVID-19) Outbreak and Pandemic Behavior | IPB |
| Kowalski et al., 2020 | Adherence to safety and self-isolation guidelines, conspiracy and paranoia-like beliefs during COVID-19 pandemic in Poland - associations and moderators | IPB |
| Oleksy et al., 2020 | Content matters. Different predictors and social consequences of general and government-related conspiracy theories on COVID-19 | IPB |
| Prati, 2020 | Intention to receive a vaccine against SARS-CoV-2 in Italy and its association with trust, worry and beliefs about the origin of the virus | VW |
| Romer & Jamieson, 2020 | Conspiracy theories as barriers to controlling the spread of COVID-19 in the US | IPB & VW |
| Salali & Uyal, 2020 | COVID-19 vaccine hesitancy is associated with beliefs on the origin of the novel coronavirus in the UK and Turkey | VW |
| Sallam et al., 2021 | High Rates of COVID-19 Vaccine Hesitancy and Its Association with Conspiracy Beliefs: A Study in Jordan and Kuwait among Other Arab Countries | VW |
| Teovanović et al., 2020 | Irrational beliefs differentially predict adherence to guidelines and pseudoscientific practices during the COVID-19 pandemic | IPB & VW |

*Table A2*. IPB: infection-preventive behavior, VW: vaccination willingness.
